# Association between fetal sex, birthweight percentile and adverse pregnancy outcome

**DOI:** 10.1111/aogs.13709

**Published:** 2019-08-30

**Authors:** Bart Jan Voskamp, Myrthe J. C. S. Peelen, Anita C. J. Ravelli, Robin van der Lee, Ben W. J. Mol, Eva Pajkrt, Wessel Ganzevoort, Brenda M. Kazemier

**Affiliations:** ^1^ Department of Obstetrics and Gynecology Amsterdam UMC University of Amsterdam Amsterdam the Netherlands; ^2^ Department of Pediatrics and Neonatology Radboud University Medical Center Nijmegen the Netherlands; ^3^ Department of Obstetrics and Gynecology Monash University Clayton Victoria Australia

**Keywords:** adverse outcome, antepartum death, intrapartum death, neonatal death, sex, small for gestational age

## Abstract

**Introduction:**

The objective was to evaluate the association between fetal sex and adverse pregnancy outcome, while correcting for fetal growth and gestational age at delivery.

**Material and methods:**

Data from the Netherlands Perinatal Registry (1999‐2010) were used. The study population comprised all white European women with a singleton delivery between 25^+0^ and 42^+6^ weeks of gestation. Fetuses with structural or chromosomal abnormalities were excluded. Outcomes were antepartum death, intrapartum/neonatal death (from onset of labor until 28 days after birth), perinatal death (antepartum death or intrapartum/neonatal death), a composite of neonatal morbidity (including infant respiratory distress syndrome, sepsis, necrotizing enterocolitis, meconium aspiration, persistent pulmonary hypertension of the newborn, periventricular leukomalacia, Apgar score <7 at 5 minutes, and intracranial hemorrhage) and a composite adverse neonatal outcome (perinatal death or neonatal morbidity). Outcomes were expressed stratified by birthweight percentile (<p10 [small for gestation], p10‐90 [normal weight], >p90 [large for gestation]) and gestational age at delivery (25^+0^‐27^+6^, 28^+0^‐31^+6^, 32^+0^‐36^+6^, 37^+0^‐42^+6 ^weeks). The association between fetal sex and outcome was assessed using the fetus at risk approach.

**Results:**

We studied 1 742 831 pregnant women. We found no increased risk of antepartum, intrapartum/neonatal and perinatal death in normal weight and large‐for‐gestation males born after 28^+0 ^weeks compared with females. We found an increased risk of antepartum death among small‐for‐gestation males born after 28^+0 ^weeks (relative risk [RR] 1.16‐1.40). All males born after 32^+0^ weeks of gestation suffered more neonatal morbidity than females regardless of birthweight percentile (RR 1.07‐1.34). Infant respiratory distress syndrome, sepsis, persistent pulmonary hypertension of the newborn, Apgar score <7 at 5 minutes, and intracranial hemorrhage all occurred more often in males than in females.

**Conclusions:**

Small‐for‐gestation males have an increased risk of antepartum death and all males born after 32^+0^ weeks of gestation have an increased risk of neonatal morbidity compared with females. In contrast to findings in previous studies we found no increased risk of antepartum, intrapartum/neonatal or perinatal death in normal weight and large‐for‐gestation males born after 28+0^ ^weeks.

**Abbreviations**
CI: confidence interval
DN: delivered neonates
ICH: intracranial hemorrhage
IND: intrapartum/neonatal death
IRDS: infant respiratory distress syndrome
LGA: large for gestational age
NEC: necrotizing enterocolitis
PERINED: Netherlands’ perinatal registry
PND: perinatal death
PPHN: persistent pulmonary hypertension of the newborn
RR: relative risk
SGA: small for gestational age

**Key message**

Small‐for‐gestational‐age males have increased risks of antepartum death; all males born after 32^+0^ weeks have increased risks of neonatal morbidity compared with females. We found no increased risk of any mortality in normal weight or large‐for‐gestational‐age males after 28^+0 ^weeks.

## 1 INTRODUCTION

In pregnancy, fetal sex is known to affect placentation,^1^ intrauterine growth,^2^ preterm birth^3^, ^4^, ^5^, ^6^, ^7^, ^8^, ^9^ and perinatal outcome.^10^, ^11^, ^12^, ^13^ Previous studies have suggested a male predominance in miscarriage,^10^ antepartum death,^11^, ^12^, ^13^ perinatal death, fetal distress, respiratory distress syndrome and low Apgar scores.^14^ Although these studies provide valuable information about gender differences, outcomes should be interpreted with care because of methodological weaknesses. First, in some studies, outcomes were adjusted for absolute birthweight and gestational age at delivery while not taking into account that healthy males are on average heavier than healthy females at any given gestational age.^2^ This may lead to comparison of small‐for‐gestation (SGA) males with normal weight females and falsely suggest a higher risk of adverse outcome among males. In other studies, outcomes were based on calculated male to female ratio for adverse outcome, expressed as the number of adverse outcomes among males divided by the number of adverse outcomes among females**.** Since, historically, more males are born, this comparison may also falsely suggest a higher risk of adverse outcome among males. Secondly, previous studies did not investigate whether there was an association between gestational age at delivery, fetal growth and perinatal outcome.

These methodological limitations can be overcome when birthweight percentiles are used to express growth instead of absolute birthweight, stratification for SGA, normal weight and large‐for‐gestational‐age (LGA) males, females and gestational age at delivery is performed, and the fetus at risk approach is used to rule out bias through unequal numbers of male and female infants.

The objective of this hypothesis‐generating study was to evaluate the association between fetal sex and adverse pregnancy outcome, including antepartum death, intrapartum/neonatal death and neonatal morbidity, whilst correcting for fetal growth and gestational age at delivery.

## 2 MATERIAL AND METHODS

This study was performed in a nationwide cohort with the use of the Netherlands Perinatal Registry (PERINED). The PERINED consists of population‐based data that contain information on pregnancies, deliveries and re‐admissions until 28 days after birth. The PERINED database is obtained by a validated linkage of three different registries: the midwifery registry, the obstetrics registry and the neonatology registry of hospital admissions.^15^, ^16^ Records are entered in the PERINED registry at the child's level. The coverage of the PERINED registry is approximately 96% of all deliveries in the Netherlands. It contains pregnancies of ≥22 weeks, gestational age and is used primarily for annual assessment of the quality indicators of obstetric care.

We included all white European women who delivered a singleton baby between 25^+0^ and 42^+6 ^weeks of gestation in The Netherlands between 1 January 1999, and 31 December 2010. We excluded all women who delivered an infant with congenital anomalies.^17^ Women of other ethnicities were excluded to avoid bias through differences in optimal weight for gestation in other groups not taken into account in the Dutch birthweight reference curves and to avoid bias through the presence of different risk profiles for adverse pregnancy outcomes among non‐white European women.

Gestational age at delivery was defined as extremely preterm (25^+0^‐27^+6 ^weeks), very preterm (28^+0^‐31^+6 ^weeks), moderate to late preterm (32^+0^‐36^+6 ^weeks) and term (37^+0^‐42^+6 ^weeks). The Dutch reference curves for birthweight by gestational age stratified for parity and sex were used.^18^ SGA was defined as a birthweight below the 10th percentile for gestation (<p10), appropriately grown for gestation (normal weight) as a birthweight between the 10th and 90th percentile (p10‐90) for gestation, and LGA as birthweight above the 90th percentile (>p90) for gestation.

Our outcome measures were antepartum death, intrapartum/neonatal death, perinatal death, a composite of neonatal morbidity, and a composite of adverse perinatal outcome. Antepartum death was defined as stillbirth that occurred between 25^+0 ^weeks gestational age and the onset of labor. Intrapartum and neonatal death were defined as death between the onset of labor and 28 days after birth. Perinatal death was defined as antepartum death, intrapartum death and neonatal death with definitions as described. The composite measure of neonatal morbidity consisted of: infant respiratory distress syndrome (IRDS), neonatal sepsis, necrotizing enterocolitis (NEC), meconium aspiration, persistent pulmonary hypertension of the newborn (PPHN), periventricular leukomalacia, Apgar score <7 at 5 minutes and intracranial hemorrhage (ICH), all as judged by the clinician within the first admission after birth. If a neonate suffered from neonatal morbidity and died within 28 days after birth, it was considered to have suffered intrapartum/neonatal death and not morbidity. The composite measure of adverse perinatal outcome consisted of the composite measure of neonatal morbidity and perinatal death.

PERINED registered demographic and obstetric characteristics including maternal age, parity, hypertensive complications of pregnancy (chronic hypertension, pregnancy‐induced hypertension, preeclampsia)^19^ and socioeconomic status. Parity was categorized into nulliparous and multiparous women. The socioeconomic status score is based on mean income level, the percentage of households with a low income, the percentage of inhabitants without a paid job and the percentage of households with on average a low education in a postal code area.^20^ The continuous socioeconomic status score was categorized into a low, middle and high group, based on percentile ranges (<25th percentile, 25‐75th percentile, >75th percentile).

Demographic and obstetric baseline characteristics were compared between males and females using Student's *t* test and Chi‐square test as appropriate. We tested for interaction between fetal sex and gestational age at delivery and between fetal sex and birthweight percentile. These tests were performed separately for all outcome measures. If interaction was found to be present (defined as a *P* < 0.001), analyses were performed, stratified for gestational age at delivery (25^+0^‐27^+6^ weeks, 28^+0^‐31^+6^ weeks, 32^+0^‐36^+6 ^weeks, 37^+0^‐42^+6 ^weeks) and birthweight percentile group (SGA, normal weight and LGA).

The fetus at risk approach was used to determine the association between fetal sex and pregnancy outcome, expressed as relative risk (RR) with 95% confidence intervals (CI) within the strata for birthweight percentile and gestational age at delivery.

### 2.1 Antepartum death

For a fetus to be at risk of antepartum death (AD) at 36^+0^ weeks it is necessary to be alive at 36^+0^ weeks. Consequently, the risk of antepartum death was calculated as a proportion of the ongoing pregnancies (OP) at a particular gestation.

Risk of antepartum death at week n = AD_n_/OP_n_*100.

### 2.2 Intrapartum/neonatal death

In concurrence, the risk of intrapartum/neonatal death (IND) at any gestational age is obtained by dividing the number of intrapartum and neonatal deaths at that gestation by the number of neonates at risk of intrapartum/neonatal death at that gestation. The neonates at risk of intrapartum/neonatal death at a certain gestational age include all delivered neonates (DN) that did not die antepartum, meaning all pregnancies with onset of labor.

Risk of intrapartum/neonatal death at week n = IND_n_/DN_n_*100.

### 2.3 Perinatal death

The risk of perinatal death (PND) at any gestational age is obtained by dividing the number of neonates with perinatal death (antepartum or intrapartum/neonatal death) in each stratum by the total number of deliveries in each birthweight category.

Risk of perinatal death at week n = PND_n_/DN_n_*100.

#### 2.3.1 Composite neonatal morbidity

The denominator used for the risk of neonatal morbidity (NM) is all alive fetuses (AF) born at that gestation, since only alive fetuses can suffer morbidity.

Risk of neonatal morbidity at week n = NM_n_/AF_n_*100.

#### 2.3.2 Composite adverse perinatal outcome

The numerator is all neonates with perinatal death (PND) or morbidity (NM) in each stratum and the denominator all deliveries in each birthweight category (DN).

Risk of adverse perinatal outcome at week n = (PND_n _+ NM_n_)/DN_n_*100.

A post‐hoc analysis for all separate components of morbidity was performed to differentiate further which outcome contributes most to the found difference between male and female neonates.

Data were analyzed with the SAS statistical software package version 9.2 (SAS Institute Inc., Cary, NC, USA).

### 2.4 Ethical approval

The data in the perinatal registry are anonymous and exempt from ethical approval. The Netherlands Perinatal Registry gave approval for the use of the data for this study (approval number 13.73).

## 3 RESULTS

From 1 January 1999 until 31 December 2010 a total of 2 078 327 singleton pregnancies between 25 and 42 weeks without congenital anomalies were identified in the PERINED database. After exclusion of non‐white European women (n = 335 426 [16%]) and infants with an unknown gender (n = 70 [0.004%]), our study population consisted of 1 742 831 pregnancies.

Baseline characteristics of the cohort are presented in Table 1. There were more male (n = 895 272 [51.4%]) than female infants (n = 847 559 [48.6%]) in the cohort. There were no statistically significant differences in maternal baseline characteristics between the two groups. However, women with a male fetus were more likely to have a hypertensive complication of pregnancy compared with women with a female fetus (9.3 vs 9.0%, *P* < 0.001). Women with a male fetus were more likely (all *P* < 0.001) than women with a female fetus to undergo an emergency cesarean section (8.5 vs 7.1%) or vaginal instrumental delivery (12.2% vs 10.0%). The rate of preterm delivery (<37 weeks of gestation) was higher among males than among females (6.4 vs 5.4%). Average birthweight in males was 124 g (95% CI 122‐126) higher than in females (3525 vs 3401 g; *P* < 0.001). Finally, male and female fetuses were equally likely to be SGA (8.9%) or LGA (11%).

**Table 1:** Characteristics of the 1 742 831 singleton white European pregnancies without congenital anomalies in the Netherlands, 1999‐2010

	Male infants	Female infants	*P* value
	(n = 895 272)	(n = 847 559)	
Maternal characteristics			
Maternal age, years (mean), (SD)	30.7 (4.6)	30.7 (4.7)	0.24
Nulliparous, n (%)	426 995 (47.7)	403 106 (47.6)	0.08
Low socioeconomic status, n (%)	172 737 (19.3)	163 006 (19.2)	0.30
Pregnancy and delivery characteristics			
Conception IVF/ICSI, n (%)	24 856 (2.8)	23 375 (2.8)	0.46
Hypertensive disorders^a^, n (%)	83 170 (9.3)	76 100 (9.0)	<0.0001
Induction of labor, n (%)	191 330 (21.4)	182 001 (21.5)	0.10
Mode of delivery			
Spontaneous vaginal delivery, n (%)	654 129 (73.1)	647 524 (76.4)	<0.0001
Instrumental vaginal delivery, n (%)	109 448 (12.2)	84 552 (10.0)	
Elective cesarean, n (%)	55 269 (6.2)	55 556 (6.6)	
Emergency cesarean, n (%)	76 426 (8.5)	59 957 (7.1)	
Neonatal characteristics			
Gestational age at delivery (wk), median (IQR)	39 (38‐40)	40 (39‐40)	<0.0001
Delivery <32 wk GA, (%)	8110 (0.9)	6438 (0.8)	<0.0001
Delivery <37 wk GA, (%)	57 353 (6.4)	45 980 (5.4)	<0.0001
Birthweight (g), mean (SD)	3525 (597)	3401 (564)	<0.0001
Birthweight <p10 (SGA)	79 442 (8.9)	75 691 (8.9)	
Birthweight p10‐p90 (normal weight)	719 424 (80.4)	677 826 (80.0)	
Birthweight ≥p90 (LGA)	96 406 (10.8)	94 042 (11.1)	

Abbreviation: SD, standard deviation.

a Preexisting hypertension, pregnancy‐induced hypertension, preeclampsia.

Interaction between fetal sex and gestational age at delivery was statistically significant for antepartum death (*P* < 0.001), intrapartum/neonatal death (*P* < 0.001) and neonatal morbidity (*P* < 0.001). Interaction between fetal sex and birthweight percentile was also significant for all outcome measures (all *P* < 0.001). Therefore, outcomes are presented stratified for four strata of gestational age at delivery (25^+0^‐27^+6^ weeks, 28^+0^‐31^+6^ weeks, 32^+0^‐36^+6 ^weeks, 37^+0^‐42^+6 ^weeks) and three strata of birthweight percentile (SGA, normal weight and LGA).

### 3.1 Antepartum death

Table 2 shows the relative risk for antepartum death separately for four strata of gestational age at delivery (25^+0^‐27^+6 ^weeks, 28^+0^‐31^+6 ^weeks, 32^+0^‐36^+6 ^weeks, 36^+6^‐42^+6 ^weeks) and three strata of birthweight percentile (SGA, normal weight and LGA).

**Table 2:** Antepartum death rate in males and females by birthweight percentiles and gestational age category

	Male infants		Female infants		RR^a^	(95% CI)
	(n = 895 272)	%	(n = 847 559)	%		
Birthweight <p10 (SGA)						
25‐27 wk GA	397/79 442	0.50	371/75 691	0.49	1.02	(0.89‐1.17)
28‐31 wk GA	303/78 863	0.38	206/75 223	0.27	**1.40**	**(1.17‐1.67)**
32‐36 wk GA	370/78 017	0.47	264/74 710	0.35	**1.34**	**(1.15‐1.57)**
37‐42 wk GA	503/73 106	0.69	420/70 774	0.59	**1.16**	**(1.02‐1.32)**
Birthweight p10‐p90 (normal weight)						
25‐27 wk GA	277/719 424	0.04	304/677 826	0.04	0.86	(0.73‐1.01)
28‐31 wk GA	404/718 042	0.06	422/676 696	0.06	0.90	(0.79‐1.03)
32‐36 wk GA	597/713 388	0.08	563/673 002	0.08	1.00	(.89‐1.12)
37‐42 wk GA	937/671 723	0.14	978/640 051	0.15	0.91	(0.83‐1.00)
Birthweight ≥p90 (LGA)						
25‐27 wk GA	31/96 406	0.03	29/94 042	0.03	1.04	(0.63‐1.73)
28‐31 wk GA	46/96 243	0.05	44/93 850	0.05	1.02	(0.67‐1.54)
32‐36 wk GA	36/95 757	0.04	24/93 409	0.03	1.46	(0.87‐2.45)
37‐42 wk GA	131/93 090	0.14	122/90 754	0.13	1.05	(0.82‐1.34)
All birthweights						
25‐27 wk GA	705/895 272	0.08	704/847 559	0.08	0.95	(0.85‐1.05)
28‐31 wk GA	753/893 148	0.08	672/845 769	0.08	1.06	(0.96‐1.18)
32‐36 wk GA	1003/887 162	0.11	851/841 121	0.10	**1.12**	**(1.02‐1.22)**
37‐42 wk GA	1571/837 919	0.19	1520/801 579	0.19	0.99	(0.92‐1.06)

Abbreviations: CI, confidence interval; LGA, large‐for‐gestational age; RR, relative risk ratio; SGA, small‐for‐gestational age.

a RR calculated with fetus at risk approach, meaning the numerator is all neonates with antepartum death in each stratum and the denominator all women remaining pregnant at the beginning of the gestational age stratum in each birthweight category.

Antepartum death occurs more often in SGA males than SGA females after 28^+0^ weeks (from 28^+0^ to 31^+6^ weeks, RR 1.40, 95% CI 1.17‐1.67; from 32^+0^ to 36^+6^ weeks, RR 1.34, 95% CI 1.15‐1.57; from 37^+0^ to 42^+6^ weeks, RR 1.16, 95% CI 1.02‐1.32).

For normal weight and LGA infants, there was no statistically significant difference between males and females. The analysis for all infants (not stratified into SGA, normal weight and LGA) shows that antepartum death occurs more often in males than in females born between 32^+0^ and 36^+6^ weeks (RR 1.12, 95% CI 1.02‐1.22).

### 3.2 Intrapartum and neonatal death

Table 3 shows the rates of intrapartum/neonatal death. In most groups, there were no significant differences between males and females. However, normal weight males born between 25^+0^ and 27^+6^ weeks GA had an increased risk of intrapartum/neonatal death compared with females (RR 1.20, 95% CI 1.02‐1.40).

**Table 3:** Intrapartum and neonatal death rate in males and females by birthweight percentiles and gestational age category

	Male infants		Female infants		RR^a^	(95% CI)
	(n = 895 272)	%	(n = 847 559)	%		
Birthweight <p10 (SGA)						
25‐27 wk GA	61/182	34	41/97	42	.79	(0.58‐1.08)
28‐31 wk GA	68/543	13	30/307	9.8	1.28	(0.85‐1.92)
32‐36 wk GA	110/4541	2.4	103/3672	2.8	0.86	(0.66‐1.13)
37‐42 wk GA	263/72 603	0.36	232/70 354	0.33	1.10	(0.92‐1.31)
Birthweight p10‐p90 (normal weight)						
25‐27 wk GA	298/1105	27	186/826	23	**1.20**	**(1.02‐1.40)**
28‐31 wk GA	203/4250	4.8	173/3272	5.3	0.90	(0.74‐1.10)
32‐36 wk GA	303/41 068	0.73	202/323 88	0.62	1.18	(0.99‐1.41)
37‐42 wk GA	549/670 786	0.08	470/639 073	0.07	1.11	(0.98‐1.26)
Birthweight ≥p90 (LGA)						
25‐27 wk GA	21/132	16	36/163	22	0.72	(0.44‐1.17)
28‐31 wk GA	33/440	7.5	31/397	7.8	0.96	(0.60‐1.54)
32‐36 wk GA	24/2631	0.91	27/2631	1.0	0.89	(0.51‐1.54)
37‐42 wk GA	64/92 959	0.07	53/90 632	0.06	1.18	(0.82‐1.69)
All birthweights						
25‐27 wk GA	380/1419	27	263/1086	24	1.08	(0.94‐1.25)
28‐31 wk GA	304/5233	5.8	234/3976	5.9	0.99	(0.84‐1.16)
32‐36 wk GA	437/48 240	0.91	332/38 691	0.86	1.06	(0.92‐1.22)
37‐42 wk GA	876/836 348	0.10	755/800 059	0.09	**1.11**	**(1.01‐1.22)**

Abbreviations: CI, confidence interval; GA, gestational age; LGA, large‐for‐gestational age; RR, relative risk ratio; SGA, small‐for‐gestational age.

a RR calculated as: the numerator is all neonates with intrapartum or neonatal death in each stratum and the denominator all deliveries without antepartum death in each birthweight category.

The analysis for all infants (not stratified into SGA, normal weight and LGA) shows that intrapartum/neonatal death occurs more often in males than in females born between 37^+0^ and 42^+6^ weeks (RR 1.11, 95% CI 1.01‐1.22).

### 3.3 Perinatal death

Table 4 shows the rates of perinatal death. In most groups, there were no significant differences between males and females. However, SGA males born between 37^+0^ and 42^+6^ weeks GA had an increased risk of perinatal death compared with females (RR 1.06, 95% CI 1.01‐1.12).

**Table 4:** Perinatal death rate in males and females by birthweight percentiles and gestational age category

	Male infants		Female infants		RR^a^	(95% CI)
	(n = 895 272)	%	(n = 847 559)	%		
Birthweight <p10 (SGA)						
25‐27 wk GA	458/579	79	412/468	88	0.77	(0.37‐0.73)
28‐31 wk GA	371/846	44	236/513	46	0.96	(0.89‐1.05)
32‐36 wk GA	480/4911	9.8	367/3936	9.3	1.02	(0.96‐1.09)
37‐42 wk GA	766/73 106	1.0	652/70 774	0.92	**1.06**	**(1.01‐1.12)**
Birthweight p10‐p90 (normal weight)						
25‐27 wk GA	575/1382	42	490/1130	43	0.97	(.90‐1.04)
28‐31 wk GA	607/4654	13	595/3694	16	0.89	(0.84‐0.95)
32‐36 wk GA	900/41 665	2.2	765/32 951	2.3	0.97	(0.92‐1.01)
37‐42 wk GA	1486/671 723	0.22	1448/640 051	0.23	0.99	(0.95‐1.03)
Birthweight ≥p90 (LGA)						
25‐27 wk GA	52/163	32	65/192	34	0.95	(0.73‐1.22)
28‐31 wk GA	79/486	16	75/441	17	0.97	(0.81‐1.15)
32‐36 wk GA	60/2667	2.2	51/2655	1.9	1.08	(0.89‐1.27)
37‐42 wk GA	195/93 090	0.21	175/90 754	0.19	1.04	(0.94‐1.14)
All birthweights						
25‐27 wk GA	1085/2124	51	967/1790	54	0.95	(0.89‐1.01)
28‐31 wk GA	1057/5986	18	906/4648	19	**0.95**	**(0.90‐0.99)**
32‐36 wk GA	1440/49 243	2.9	1183/39 542	3.0	0.99	(0.95‐1.03)
37‐42 wk GA	2447/837 919	0.29	2275/801 579	0.28	1.01	(0.99‐1.04)

Abbreviations: CI, confidence interval; GA, gestational age; LGA, large‐for‐gestational age; RR, relative risk ratio; SGA, small‐for‐gestational age.

a RR calculated as: the numerator are all neonates with perinatal death (antepartum or intrapartum/neonatal death) in each stratum and the denominator all deliveries in each birthweight category.

In contrast, the analysis for all infants (not stratified into SGA, normal weight and LGA) shows that perinatal death occurs less often in males than in females born between 28^+0^ and 31^+6^ weeks (RR 0.95, 95% CI 0.90‐0.99).

### 3.4 Composite morbidity

The composite morbidity of males and females is shown in Table 5.

**Table 5:** Composite neonatal morbidity rate in males and females by birthweight percentiles and gestational age category

	Male infants		Female infants		RR^a^	(95% CI)
	(n = 895 272)	%	(n = 847 559)	%		
Birthweight <p10 (SGA)						
25‐27 wk GA	94/121	78	48/56	86	0.91	(0.78‐1.05)
28‐31 wk GA	324/475	68	190/277	69	0.99	(0.90‐1.10)
32‐36 wk GA	535/4431	12	366/3569	10	**1.18**	**(1.04‐1.33)**
37‐42 wk GA	1723/72 340	2.4	1320/70 122	1.9	**1.27**	**(1.18‐1.36)**
Birthweight p10‐p90 (normal weight)						
25‐27 wk GA	674/807	84	527/807	82	1.01	(0.97‐1.06)
28‐31 wk GA	2602/4047	64	1861/3099	60	**1.07**	**(1.03‐1.11)**
32‐36 wk GA	3754/40 765	9.2	2550/32 186	7.9	**1.16**	**(1.11‐1.22)**
37‐42 wk GA	9292/670 237	1.4	6533/638 603	1.0	**1.36**	**(1.31‐1.40)**
Birthweight ≥p90 (LGA)						
25‐27 wk GA	87/111	78	105/127	83	0.95	(0.84‐1.08)
28‐31 wk GA	248/407	60	197/366	54	**1.13**	**(1.00‐1.28)**
32‐36 wk GA	274/2607	11	213/2604	8.2	**1.28**	**(1.08‐1.52)**
37‐42 wk GA	1698/92 895	1.8	1233/90 579	1.4	**1.34**	**(1.25‐1.44)**
All birthweights						
25‐27 wk GA	855/1039	82	680/823	83	0.99	(0.95‐1.04)
28‐31 wk GA	3174/4929	64	2248/3742	60	**1.07**	**(1.04‐1.11)**
32‐36 wk GA	4563/47 803	9.5	3129/38 359	8.2	**1.17**	**(1.12‐1.22)**
37‐42 wk GA	12 713/835 472	1.5	9086/799 304	1.1	**1.34**	**(1.30‐1.37)**

Abbreviations: CI, confidence interval; GA, gestational age; LGA, large‐for‐gestational age; RR, relative risk ratio; SGA, small‐for‐gestational age.

a RR calculated as: the numerator is all alive neonates with morbidity in each stratum and the denominator all alive neonates in each birthweight category.

The risk of composite morbidity was significantly increased in males compared with females in most (8/12) strata, with relative risks ranging from 1.07 to 1.36. All males born after 32^+0^ weeks of gestation have an increased risk of neonatal morbidity compared with females.

The analysis for all infants (not stratified into SGA, normal weight and LGA) shows that composite morbidity was significantly increased in males compared with females born after 28^+0^ weeks, with relative risks ranging from 1.07 to 1.34.

### 3.5 Composite adverse perinatal outcome

The composite adverse neonatal outcome of males and females is shown in Table 6.

**Table 6:** Composite adverse perinatal outcome rate in males and females by birthweight percentiles and gestational age category

	Male infants		Female infants		RR^a^	(95% CI)
	(n = 895 272)	%	(n = 847 559)	%		
Birthweight <p10 (SGA)						
25‐27 wk GA	552/579	95	460/468	98	0.97	(0.95‐0.99)
28‐31 wk GA	695/846	82	426/513	83	0.99	(0.94‐1.04)
32‐36 wk GA	1015/4911	21	733/3936	19	**1.11**	**(1.02‐1.21)**
37‐42 wk GA	2489/73 106	3.4	1972/70 774	2.8	**1.22**	**(1.15‐1.30)**
Birthweight p10‐p90 (normal weight)						
25‐27 wk GA	1249/1382	90	1017/1130	90	1.00	(0.98‐1.03)
28‐31 wk GA	3209/4654	69	2456/3694	66	**1.04**	**(1.01‐1.07)**
32‐36 wk GA	4654/41 665	11	3315/32 951	10	**1.11**	**(1.06‐1.16)**
37‐42 wk GA	10 778/671 723	1.6	7981/640 051	1.2	**1.29**	**(1.25‐1.32)**
Birthweight ≥p90 (LGA)						
25‐27 wk GA	139/163	85	170/192	89	0.96	(0.89‐1.05)
28‐31 wk GA	327/486	67	272/441	62	1.09	(0.99‐1.21)
32‐36 wk GA	334/2667	13	264/2655	10	**1.26**	**(1.08‐1.47)**
37‐42 wk GA	1893/93 090	2.0	1408/90 754	1.6	**1.31**	**(1.22‐1.40)**
All birthweights						
25‐27 wk GA	1940/2124	91	1647/1790	92	0.99	(0.97‐1.01)
28‐31 wk GA	4231/5986	71	3154/4648	68	**1.04**	**(1.02‐1.07)**
32‐36 wk GA	6003/49 243	12	4312/39 542	11	**1.12**	**(1.08‐1.16)**
37‐42 wk GA	15 160/837 919	1.8	11 361/801 579	1.4	**1.28**	**(1.25‐1.31)**

Abbreviations: CI, confidence interval; GA, gestational age; LGA, large‐for‐gestational age; RR, relative risk ratio; SGA, small‐for‐gestational age.

a RR calculated as: the numerator are all neonates with perinatal death (antepartum or intrapartum/neonatal death) or morbidity in each stratum and the denominator all deliveries in each birthweight category.

The risk of composite adverse neonatal outcome was significantly increased in males compared with females in most (7/12) strata, with relative risks ranging from 1.04 to 1.31. All males born after 32^+0^ weeks of gestation have an increased risk of adverse neonatal outcome compared with females.

The analysis for all infants (not stratified into SGA, normal weight and LGA) shows that composite morbidity was significantly increased in males compared with females born after 28^+0^ weeks, with relative risks ranging from 1.04 to 1.28.

The individual components of the composite neonatal morbidity (IRDS, sepsis, NEC, meconium aspiration, PPHN, periventricular leukomalacia, Apgar score <7 at 5 minutes and ICH) were also analyzed separately (Tables S1‐S8). IRDS was increased in males compared with females at most gestational ages, regardless of birthweight percentile (Table S1). Sepsis was increased in all term males compared with females regardless of birthweight. Between 32^+0^ and 36^+6^ weeks the risk of sepsis was only increased in normal weight males (Table S2). PPHN was only increased in normal weight males at term compared with females (Table S5). For periventricular leukomalacia, although in only one stratum, a decreased risk was found for SGA males between 25^+0^ and 27^+0^ weeks of gestation (Table S6). The risk of ICH was increased in all normal weight males compared with females regardless of gestational age at delivery and in LGA males born between 28^+0^ and 31^+6^ weeks of gestation (RR 1.52, 95% CI 1.08‐2.13) (Table S6). The incidence of an Apgar score <7 at 5 minutes was increased in all term males compared with females regardless of birthweight. Furthermore, SGA males between 25^+0^ and 27^+0^ and 32^+0^ and 36^+6^ weeks of gestation were at increased risk (Table S7). The incidence of NEC (Table S3) and meconium aspiration (Table S4) was not significantly different between males and females.

## 4 DISCUSSION

In our study, we analyzed 1 742 831 singleton deliveries and assessed differences in pregnancy outcomes between males and females after adjustments for differences in fetal growth. SGA males have an increased risk of antepartum death and all males born after 32^+0^ weeks of gestation have an increased risk of neonatal morbidity compared with females. Differences in neonatal morbidity are mainly caused by increased risks of sepsis, IRDS and ICH in males compared with females.

Our study has some limitations. These limitations are mainly related to the fact that we performed a database study and consequently had to rely on information that was recorded in the registry. To assess differences in the incidence of adverse outcome, large numbers are needed. In our opinion, it would not have been feasible to collect enough data using a different study design.

Secondly, the Dutch Perinatal registry did not contain reliable data on the use of betamethasone, maternal smoking, diabetes or maternal body mass index because these data were not mandatory fields in the perinatal registration during the study period, and as a result not reliably registered. All these factors are associated with adverse pregnancy outcome, but it is unlikely that there is a fetal sex‐based bias. Betamethasone administration is based on gestational age and is not sex‐dependent, and no publications about a relation between maternal smoking, diabetes or BMI and fetal sex could be retrieved. Adverse neonatal outcome could not be more clearly defined than that judged by the clinician within the first admission after birth, therefore we do not expect a fetal sex‐based bias. In addition, there are probably unknown factors causally related to perinatal outcome, such as maternal smoking and hypertensive disorders. By adjusting for birthweight percentile it is possible that our results are partly distorted by a biasing path through the unmeasured factors.^21^

Women with a male fetus were more likely to have a hypertensive complication of pregnancy compared with women with a female fetus (9.3 vs 9.0%, *P* < 0.001). This is in accordance with findings in previous research.^22^ The association might be term‐dependent, but this is outside the scope of this study.

The PERINED database does not contain quantitative data on the method of pregnancy dating. According to the ruling guideline during the study period, pregnancy dating was predominantly performed by first trimester ultrasound measurements (crown‐rump length). If no first trimester dating was performed, dating was based on head‐circumference measurement or last menstrual period. In the rare cases when dating was done in the second trimester, a sex‐dependent difference in estimation, since boys are already somewhat larger than girls on average at that time^23^ may disturb these analyses. Because the majority of ultrasound dating was done in the first trimester we do not expect a substantial influence on our results. Also, in case of antepartum death—especially in the preterm period—the gestational age of delivery is not the same as the moment of fetal demise. Although this might cause a structural underestimation of birthweight percentile, it is unlikely that this time‐effect is different between males and females. Also, the difference between fetal demise and delivery and thus possible overestimation of SGA in case of antepartum death is likely small at term, because—according to the Dutch protocol—all pregnant women in the term period undergo weekly checkups including Doppler auscultation of the fetal heart rate.

We used population‐based birthweight percentiles.^18^ Individual growth potential and placental characteristics might have enabled more accurate prediction of growth restriction and adverse outcome.^24^, ^25^ We were not able to correct for this because maternal length and weight, and placental weight and pathology are not registered in the Dutch Perinatal Registry. We do not expect a systematical bias.

Furthermore, ultrasound growth charts used to detect SGA fetuses are not gender‐specific. Therefore it is possible that girls (who are by definition smaller than male fetuses) are more often SGA compared with boys, perhaps leading to earlier intervention (induction) and thereby preventing antepartum death. This possible bias cannot be ruled out and should be investigated in further studies.

Our data did not allow us to make statements about the influence of mode of delivery on neonatal death and morbidity. Table 1 shows differences in induction of labor and instrumental deliveries between males and females. These are often a result of suspected compromised fetal condition rather than a confounder of adverse outcome. We can only hypothesize that the higher rate of vaginal instrumental delivery among males might be associated with the increased risk of ICH among males; another explanation could be that the ICH occurred antenatally and caused fetal distress. This is not within the scope of this paper and should be investigated in future studies. Finally, there might be an association between the higher incidence of cesarean sections among males and the risk of IRDS, although information on timing of the cesarean section and administration of betamethasone is lacking.

Finally, some causes of neonatal morbidity are predominantly a problem in the extreme preterm period, whereas others occur at all gestational ages or mainly at term. We decided to use a composite outcome containing important causes of neonatal morbidity available in the PERINED registry to provide useful information for clinicians. Stratification into four categories of gestational ages at delivery ensures that an automatic distinction is made between causes of morbidity that predominantly occur among extremely preterm infants and causes of morbidity that occur at all gestational ages. Separate tables for all causes of morbidity are provided as Tables S1‐S8.

The main strength of this study is the size (1 742 831 pregnancies) and composition of the cohort. Data are derived from a large, well‐maintained population‐based national perinatal registry (1999‐2010). Cohorts of all previous studies were smaller (549 up to 469 152 pregnancies)^11^, ^25^ and often included anomalous fetuses and/or twins. The proportion of male infants, the incidence of antepartum deaths, intrapartum/neonatal deaths and composite neonatal morbidity that we found in this study are in accordance with previous research.^7^, ^14^, ^26^, ^27^, ^28^, ^29^, ^30^

Secondly, as discussed in the introduction, previous studies falsely suggest a higher risk of adverse outcome among males because the absolute number of adverse outcomes among males was divided by the number of adverse outcomes among females**.** Using the fetus at risk approach avoids bias through unequal numbers of male and female fetuses at a certain gestational age. Also—in contrast to previous studies—outcomes were stratified for birthweight percentile and gestational age at delivery. This enables comparison of male and female infants within the same birthweight percentile category and gestational age group, and rules out bias through the association between male sex and premature delivery. As a result, the outcomes most closely represent the influence of fetal sex on the outcomes of interest and allowed us to check for associations between fetal growth, gender and adverse outcome.

To our knowledge this is the first study that tested for interaction and consequently performed analyses stratified for gestational age at delivery and birthweight percentile.

This was a hypothesis‐generating study. Unknown or unmeasured factors may have caused these associations. After appropriate correction for birthweight percentile and gestational age, the results of this study put previous studies in perspective that showed increased antepartum death, intrapartum/neonatal death and neonatal morbidity in males compared with females.^10^, ^11^, ^12^, ^13^, ^14^ These higher adverse outcome rates might not be caused by actual increased chances of adverse outcome among males but by the increased risk of preterm delivery in males,^3^, ^4^, ^5^, ^6^, ^7^, ^8^, ^9^ by adjustment for absolute birthweight instead of birthweight percentile, and calculation of male/female ratios of adverse outcome that was not adjusted for differences in the total number of male and female infants born. In contrast to previous studies, we found no increased risk of antepartum, intrapartum/neonatal and perinatal death in normal weight and LGA males born after 28^+0 ^weeks. We found an increased risk of antepartum death (after 28^+0 ^weeks) and neonatal morbidity (after 32^+0^ weeks) among SGA males, and only increased risk in perinatal death among SGA males born between 37^+0^ and 42^+6 ^weeks.

This study shows increased composite morbidity and composite adverse perinatal outcome among all males born after 32^+0^ weeks of gestation. The higher neonatal morbidity rate in males seems to be mainly caused by higher rates of IRDS, sepsis and ICH in males. The effect of higher neonatal morbidity on long‐term health outcomes in males in our cohort is unsure. Previous research showed higher rates of bronchopulmonary dysplasia and increased mortality in very preterm SGA infants and very low birthweight infants.^31^, ^32^

We hypothesize that genetic differences between males and females might underlie the fetal sex‐related differences. This study gives no clues on how to decrease neonatal morbidity in male infants.

The results of this study matter because they correct the erroneous idea that all male infants suffer more antepartum, intrapartum/neonatal and perinatal death than female infants. The main implication of this study is awareness of differences between male and female perinatal outcome. Our study has further focused on which domain these differences lie.

Further intervention studies should be aware of sex differences in perinatal outcome and should perform prespecified analyses separately for male and female infants. The importance of this advice has already been shown in a recent intervention study that found differences in effectiveness of allopurinol between male and female fetuses.^33^ This could lead to a more tailored approach fit for the individual neonate to improve outcomes. In addition, future research could be aimed at unraveling mechanisms that might play a role in the increased neonatal morbidity in males.

## 5 CONCLUSION

SGA males have an increased risk of antepartum death and all males born after 32^+0^ weeks of gestation have an increased risk of neonatal morbidity compared with females. Differences in neonatal morbidity are mainly caused by increased risks of IRDS, sepsis, PPHN, Apgar score <7 at 5 minutes and ICH in males compared with females. In contrast to findings in previous studies, we found no increased risk of antepartum, intrapartum/neonatal and perinatal death in normal weight and LGA males born after 28^+0 ^weeks.

## CONFLICT OF INTEREST

B.W.M. reports consultancy for ObsEva, Merck and Guerbet. B.W.M. is supported by a NHMRC Practitioner Fellowship (GNT1082548).
