# Supplementary material for: Association between fetal sex, birthweight percentile and adverse pregnancy outcome
Source: Acta Obstet Gynecol Scand. 2019 Aug 30;99(1):48–58. doi: 10.1111/aogs.13709 (PMC6973256; doi:10.1111/aogs.13709)
Supplement: Supplementary file 1 [file AOGS-99-48-s001.docx]

| **Table S1.** Infant respiratory distress syndrome rate in males and females by birthweight percentiles and gestational age category. | | | | | | | | | | |
| --- | --- | --- | --- | --- | --- | --- | --- | --- | --- | --- |
|  | Male infants |  | Female infants |  |  |  |  |  |  |  |
|  | (n=895 272) | % | (n=847 559) | % | RR^a^ |  | (95%CI) | | |  |
| Birthweight <p10 (SGA) | |  |  |  |  |  |  |  |  |  |
| 25-27 weeks GA | 67/121 | 55 | 38/56 | 68 | 0.82 | ( | 0.64 | - | 1.04 | ) |
| 28-31 weeks GA | 188/475 | 40 | 84/277 | 30 | **1.31** | **(** | **1.06** | **-** | **1.61** | **)** |
| 32-36 weeks GA | 133/4431 | 3.0 | 53/3569 | 1.5 | **2.02** | **(** | **1.47** | **-** | **2.77** | **)** |
| 37-42 weeks GA | 40/72 340 | 0.06 | 24/70 122 | 0.03 | 1.62 | ( | 0.97 | - | 2.68 | ) |
| Birthweight p10-p90 (normal weight) | |  |  |  |  |  |  |  |  |  |
| 25-31 weeks GA | 473/807 | 59 | 373/640 | 58 | 1.01 | ( | 0.92 | - | 1.10 | ) |
| 28-31 weeks GA | 1755/4047 | 43 | 1154/3 099 | 37 | **1.16** | **(** | **1.10** | **-** | **1.23** | **)** |
| 32-36 weeks GA | 1730/40 765 | 4.2 | 1047/32 186 | 3.3 | **1.30** | **(** | **1.21** | **-** | **1.41** | **)** |
| 37-42 weeks GA | 239/670 237 | 0.04 | 133/638 603 | 0.02 | **1.71** | **(** | **1.39** | **-** | **2.12** | **)** |
| Birthweight ≥p90 (LGA) | |  |  |  |  |  |  |  |  |  |
| 25-31 weeks GA | 56/111 | 50 | 70/127 | 55 | 0.92 | ( | 0.72 | - | 1.17 | ) |
| 28-31 weeks GA | 154/407 | 37 | 117/366 | 32 | 1.18 | ( | 0.97 | - | 1.44 | ) |
| 32-36 weeks GA | 132/2607 | 5.1 | 81/2604 | 3.1 | **1.63** | **(** | **1.24** | **-** | **2.13** | **)** |
| 37-42 weeks GA | 36/92 895 | 0.04 | 34/90 579 | 0.04 | 1.03 | ( | 0.65 | - | 1.65 | ) |
| Total | 5003/889 243 | 0.56 | 3208/842 228 | 0.38 | **1.48** | **(** | **1.41** | **-** | **1.54** | **)** |
| ^a^RR calculated with fetus at risk approach, meaning the numerator is all alive neonates with morbidity in each stratum and the denominator all alive neonates in each birthweight category  GA, gestational age; SGA, small for gestational age; LGA, large for gestational age; RR, relative risk ratio; CI, confidence interval | | | | | | | | | | |

| **Table S2.** Sepsis rate in males and females by birthweight percentiles and gestational age category. | | | | | | | | | | |
| --- | --- | --- | --- | --- | --- | --- | --- | --- | --- | --- |
|  | Male infants |  | Female infants |  |  |  |  |  |  |  |
|  | (n=895 272) | % | (n=847 559) | % | RR^a^ |  | (95%CI) | | |  |
| Birthweight <p10 (SGA) | |  |  |  |  |  |  |  |  |  |
| 25-27 weeks GA | 58/121 | 48 | 37/56 | 66 | 0.73 | ( | 0.56 | - | 0.94 | ) |
| 28-31 weeks GA | 196/475 | 41 | 109/277 | 39 | 1.05 | ( | 0.87 | - | 1.26 | ) |
| 32-36 weeks GA | 247/4431 | 5.6 | 197/3569 | 5.5 | 1.01 | ( | 0.84 | - | 1.21 | ) |
| 37-42 weeks GA | 512/72 340 | 0.71 | 402/70 122 | 0.57 | **1.23** | **(** | **1.08** | **-** | **1.41** | **)** |
| Birthweight p10-p90 (normal weight) | |  |  |  |  |  |  |  |  |  |
| 25-31 weeks GA | 424/807 | 53 | 334/640 | 52 | 1.01 | ( | 0.91 | - | 1.11 | ) |
| 28-31 weeks GA | 1234/4047 | 30 | 910/3099 | 29 | 1.04 | ( | 0.97 | - | 1.12 | ) |
| 32-36 weeks GA | 1526/40 765 | 3.7 | 1031/32 186 | 3.2 | **1.17** | **(** | **1.08** | **-** | **1.26** | **)** |
| 37-42 weeks GA | 3620/670 237 | 0.54 | 2434/638 603 | 0.38 | **1.42** | **(** | **1.35** | **-** | **1.49** | **)** |
| Birthweight ≥p90 (LGA) | |  |  |  |  |  |  |  |  |  |
| 25-31 weeks GA | 55/111 | 50 | 63/127 | 50 | 1.00 | ( | 0.77 | - | 1.29 | ) |
| 28-31 weeks GA | 118/407 | 29 | 96/366 | 26 | 1.11 | ( | 0.88 | - | 1.39 | ) |
| 32-36 weeks GA | 124/2607 | 4.8 | 99/2604 | 3.8 | 1.25 | ( | 0.97 | - | 1.62 | ) |
| 37-42 weeks GA | 707/92 895 | 0.76 | 539/90 579 | 0.60 | **1.28** | **(** | **1.14** | **-** | **1.43** | **)** |
| Total | 8821/889 243 | 0.99 | 6251/842 228 | 0.74 | **1.34** | **(** | **1.29** | **-** | **1.38** | **)** |
| ^a^RR calculated with fetus at risk approach, meaning the numerator is all alive neonates with morbidity in each stratum and the denominator all alive neonates in each birthweight category  GA, gestational age; SGA, small for gestational age; LGA, large for gestational age; RR, relative risk ratio; CI, confidence interval | | | | | | | | | | |

| **Table S3.** Necrotizing enterocolitis rate in males and females by birthweight percentiles and gestational age category. | | | | | | | | | | |
| --- | --- | --- | --- | --- | --- | --- | --- | --- | --- | --- |
|  | Male infants |  | Female infants |  |  |  |  |  |  |  |
|  | (n=895 272) | % | (n=847 559) | % | RR^a^ |  | (95%CI) | | |  |
| Birthweight <p10 (SGA) | |  |  |  |  |  |  |  |  |  |
| 25-27 weeks GA | 9/121 | 7.4 | 9/56 | 16 | 0.46 | ( | 0.19 | - | 1.10 | ) |
| 28-31 weeks GA | 43/475 | 9.1 | 27/277 | 9.7 | 0.93 | ( | 0.59 | - | 1.47 | ) |
| 32-36 weeks GA | 40/4431 | 0.90 | 32/3569 | 0.90 | 1.01 | ( | 0.63 | - | 1.60 | ) |
| 37-42 weeks GA | 11/72 340 | 0.02 | 14/70 122 | 0.02 | 0.76 | ( | 0.35 | - | 1.68 | ) |
| Birthweight p10-p90 (normal weight) | |  |  |  |  |  |  |  |  |  |
| 25-31 weeks GA | 62/807 | 7.7 | 60/640 | 9.4 | 0.82 | ( | 0.58 | - | 1.15 | ) |
| 28-31 weeks GA | 92/4047 | 2.3 | 94/3099 | 3.0 | 0.75 | ( | 0.56 | - | 1.00 | ) |
| 32-36 weeks GA | 74/40 765 | 0.18 | 60/32 186 | 0.19 | 0.97 | ( | 0.69 | - | 1.37 | ) |
| 37-42 weeks GA | 28/670 237 | 0.004 | 17/638 603 | 0.003 | 1.57 | ( | 0.86 | - | 2.87 | ) |
| Birthweight ≥p90 (LGA) | |  |  |  |  |  |  |  |  |  |
| 25-31 weeks GA | 7/111 | 6.3 | 8/127 | 6.3 | 1.00 | ( | 0.38 | - | 2.67 | ) |
| 28-31 weeks GA | 3/407 | 0.74 | 3/366 | 0.82 | 0.90 | ( | 0.18 | - | 4.43 | ) |
| 32-36 weeks GA | 4/2607 | 0.15 | 6/2604 | 0.23 | 0.67 | ( | 0.19 | - | 2.36 | ) |
| 37-42 weeks GA | 4/92 895 | 0.004 | 2/90 579 | 0.002 | 1.95 | ( | 0.36 | - | 10.65 | ) |
| Total | 377/889 243 | 0.04 | 332/842 228 | 0.04 | 1.08 | ( | 0.93 | - | 1.25 | ) |
| ^a^RR calculated with fetus at risk approach, meaning the numerator is all alive neonates with morbidity in each stratum and the denominator all alive neonates in each birthweight category  GA, gestational age; SGA, small for gestational age; LGA, large for gestational age; RR, relative risk ratio; CI, confidence interval | | | | | | | | | | |

| **Table S4.** Meconium aspiration rate in males and females by birthweight percentiles and gestational age category. | | | | | | | | | |  |
| --- | --- | --- | --- | --- | --- | --- | --- | --- | --- | --- |
|  | Male infants |  | Female infants |  |  |  |  |  |  |  |
|  | (n=895 272) | % | (n=847 559) | % | RR^a^ |  | (95%CI) | | |  |
| Birthweight <p10 (SGA) | |  |  |  |  |  |  |  |  |  |
| 25-27 weeks GA | 1/121 | 0.83 | 0/56 | 0 | NA | ( |  | - |  | ) |
| 28-31 weeks GA | 0/475 | 0 | 0/277 | 0 | NA | ( |  | - |  | ) |
| 32-36 weeks GA | 1/4431 | 0.02 | 0/3569 | 0 | NA | ( |  | - |  | ) |
| 37-42 weeks GA | 105/72 340 | 0.15 | 109/70 122 | 0.16 | 0.93 | ( | 0.71 | - | 1.22 | ) |
| Birthweight p10-p90 (normal weight) | |  |  |  |  |  |  |  |  |  |
| 25-31 weeks GA | 2/807 | 0.25 | 0/640 | 0 | NA | ( |  | - |  | ) |
| 28-31 weeks GA | 1/4047 | 0.02 | 1/3099 | 0.03 | 0.77 | ( | 0.05 | - | 12.24 | ) |
| 32-36 weeks GA | 7/40765 | 0.02 | 3/32 186 | 0.009 | 1.84 | ( | 0.48 | - | 7.12 | ) |
| 37-42 weeks GA | 577/670 237 | 0.09 | 507/638 603 | 0.08 | 1.08 | ( | 0.96 | - | 1.22 | ) |
| Birthweight ≥p90 (LGA) | |  |  |  |  |  |  |  |  |  |
| 25-31 weeks GA | 0/111 | 0 | 0/127 | 0 | NA | ( |  | - |  | ) |
| 28-31 weeks GA | 0/407 | 0 | 1/366 | 0.27 | NA | ( |  | - |  | ) |
| 32-36 weeks GA | 1/2607 | 0.04 | 0/2604 | 0 | NA | ( |  | - |  | ) |
| 37-42 weeks GA | 97/92 895 | 0.10 | 71/90 579 | 0.08 | 1.33 | ( | 0.98 | - | 1.81 | ) |
| Total | 792/889 243 | 0.09 | 692/842 228 | 0.08 | 1.08 | ( | 0.98 | - | 1.20 | ) |
| ^a^RR calculated with fetus at risk approach, meaning the numerator is all alive neonates with morbidity in each stratum and the denominator all alive neonates in each birthweight category  GA, gestational age; SGA, small for gestational age; LGA, large for gestational age; RR, relative risk ratio; CI, confidence interval | | | | | | | | | | |

| **Table S5.** Persistent pulmonary hypertension of the newborn rate in males and females by birthweight percentiles and gestational age category. | | | | | | | | | | |
| --- | --- | --- | --- | --- | --- | --- | --- | --- | --- | --- |
|  | Male infants |  | Female infants |  |  |  |  |  |  |  |
|  | (n=895 272) | % | (n=847 559) | % | RR^a^ |  | (95%CI) | | |  |
| Birthweight <p10 (SGA) | |  |  |  |  |  |  |  |  |  |
| 25-27 weeks GA | 4/121 | 3.3 | 1/56 | 1.8 | 1.85 | ( | 0.21 | - | 16.19 | ) |
| 28-31 weeks GA | 5/475 | 1.1 | 2/277 | 0.72 | 1.46 | ( | 0.28 | - | 7.46 | ) |
| 32-36 weeks GA | 6/4431 | 0.14 | 6/3569 | 0.17 | 0.81 | ( | 0.26 | - | 2.50 | ) |
| 37-42 weeks GA | 36/72 340 | 0.05 | 33/70 122 | 0.05 | 1.06 | ( | 0.66 | - | 1.70 | ) |
| Birthweight p10-p90 (normal weight) | |  |  |  |  |  |  |  |  |  |
| 25-31 weeks GA | 16/807 | 2.0 | 14/640 | 2.2 | 0.91 | ( | 0.45 | - | 1.84 | ) |
| 28-31 weeks GA | 46/4047 | 1.1 | 37/3099 | 1.2 | 0.95 | ( | 0.62 | - | 1.46 | ) |
| 32-36 weeks GA | 65/40 765 | 0.16 | 45/32 186 | 0.14 | 1.14 | ( | 0.78 | - | 1.67 | ) |
| 37-42 weeks GA | 199/670 237 | 0.03 | 129/638 603 | 0.02 | **1.47** | **(** | **1.18** | **-** | **1.83** | **)** |
| Birthweight ≥p90 (LGA) | |  |  |  |  |  |  |  |  |  |
| 25-31 weeks GA | 3/111 | 2.7 | 3/127 | 2.4 | 1.14 | ( | 0.24 | - | 5.55 | ) |
| 28-31 weeks GA | 1/407 | 0.25 | 1/366 | 0 | NA | ( |  | - |  | ) |
| 32-36 weeks GA | 7/2607 | 0.27 | 4/2604 | 0.15 | 1.75 | ( | 0.51 | - | 5.96 | ) |
| 37-42 weeks GA | 39/92 895 | 0.04 | 30/90 579 | 0.03 | 1.27 | ( | 0.79 | - | 2.04 | ) |
| Total | 427/888 816 | 0.05 | 304/841 924 | 0.04 | **1.33** | **(** | **1.15** | **-** | **1.54** | **)** |
| ^a^RR calculated with fetus at risk approach, meaning the numerator is all alive neonates with morbidity in each stratum and the denominator all alive neonates in each birthweight category  GA, gestational age; SGA, small for gestational age; LGA, large for gestational age; RR, relative risk ratio; CI, confidence interval | | | | | | | | | | |

| **Table S6.** Periventricular leukomalacia rate in males and females by birthweight percentiles and gestational age category. | | | | | | | | | | |
| --- | --- | --- | --- | --- | --- | --- | --- | --- | --- | --- |
|  | Male infants |  | Female infants |  |  |  |  |  |  |  |
|  | (n=895 272) | % | (n=847 559) | % | RR^a^ |  | (95%CI) | | |  |
| Birthweight <p10 (SGA) | |  |  |  |  |  |  |  |  |  |
| 25-27 weeks GA | 8/121 | 6.6 | 10/56 | 18 | **0.37** | **(** | **0.15** | **-** | **0.89** | **)** |
| 28-31 weeks GA | 38/475 | 8.0 | 23/277 | 8.3 | 0.96 | ( | 0.59 | - | 1.58 | ) |
| 32-36 weeks GA | 16/4431 | 0.36 | 19/3569 | 0.53 | 0.68 | ( | 0.35 | - | 1.32 | ) |
| 37-42 weeks GA | 11/72 340 | 0.02 | 3/70 122 | 0.004 | 3.55 | ( | 0.99 | - | 12.74 | ) |
| Birthweight p10-p90 (normal weight) | |  |  |  |  |  |  |  |  |  |
| 25-31 weeks GA | 70/807 | 8.7 | 49/640 | 7.7 | 1.13 | ( | 0.80 | - | 1.61 | ) |
| 28-31 weeks GA | 220/4047 | 5.4 | 150/3099 | 4.8 | 1.12 | ( | 0.92 | - | 1.37 | ) |
| 32-36 weeks GA | 77/40 765 | 0.19 | 45/32 186 | 0.14 | 1.35 | ( | 0.94 | - | 1.95 | ) |
| 37-42 weeks GA | 15/670 237 | 0.002 | 12/638 603 | 0.002 | 1.19 | ( | 0.56 | - | 2.54 | ) |
| Birthweight ≥p90 (LGA) | |  |  |  |  |  |  |  |  |  |
| 25-31 weeks GA | 9/111 | 8.1 | 10/127 | 7.9 | 1.03 | ( | 0.43 | - | 2.44 | ) |
| 28-31 weeks GA | 17/407 | 4.2 | 17/366 | 4.6 | 0.90 | ( | 0.47 | - | 1.74 | ) |
| 32-36 weeks GA | 3/2607 | 0.12 | 4/2604 | 0.15 | 0.75 | ( | 0.17 | - | 3.34 | ) |
| 37-42 weeks GA | 1/92 895 | 0.001 | 2/90 579 | 0.002 | 0.49 | ( | 0.04 | - | 5.38 | ) |
| Total | 485/889 243 | 0.05 | 344/842 228 | 0.04 | **1.34** | **(** | **1.16** | **-** | **1.53** | **)** |
| ^a^RR calculated with fetus at risk approach, meaning the numerator is all alive neonates with morbidity in each stratum and the denominator all alive neonates in each birthweight category  GA, gestational age; SGA, small for gestational age; LGA, large for gestational age; RR, relative risk ratio; CI, confidence interval | | | | | | | | | | |

| **Table S7.** Apgar score <7 at 5 minutes rate in males and females by birthweight percentiles and gestational age category. | | | | | | | | | | |
| --- | --- | --- | --- | --- | --- | --- | --- | --- | --- | --- |
|  | Male infants |  | Female infants |  |  |  |  |  |  |  |
|  | (n=895 272) | % | (n=847 559) | % | RR^a^ |  | (95%CI) | | |  |
| Birthweight <p10 (SGA) | |  |  |  |  |  |  |  |  |  |
| 25-27 weeks GA | 33/121 | 27 | 7/56 | 13 | **2.18** | **(** | **1.03** | **-** | **4.63** | **)** |
| 28-31 weeks GA | 45/475 | 9.5 | 37/277 | 13 | 0.71 | ( | 0.47 | - | 1.07 | ) |
| 32-36 weeks GA | 173/4431 | 3.9 | 103/3569 | 2.9 | **1.35** | **(** | **1.06** | **-** | **1.72** | **)** |
| 37-42 weeks GA | 1154/72 340 | 1.6 | 855/70 122 | 1.2 | **1.31** | **(** | **1.20** | **-** | **1.43** | **)** |
| Birthweight p10-p90 (normal weight) | |  |  |  |  |  |  |  |  |  |
| 25-31 weeks GA | 197/807 | 24 | 158/640 | 25 | 0.99 | ( | 0.82 | - | 1.19 | ) |
| 28-31 weeks GA | 495/4047 | 12 | 360/3099 | 12 | 1.05 | ( | 0.93 | - | 1.20 | ) |
| 32-36 weeks GA | 999/40 765 | 2.5 | 722/32 186 | 2.2 | 1.09 | ( | 0.99 | - | 1.20 | ) |
| 37-42 weeks GA | 5300/670 237 | 0.79 | 3839/638 603 | 0.60 | **1.32** | **(** | **1.26** | **-** | **1.37** | **)** |
| Birthweight ≥p90 (LGA) | |  |  |  |  |  |  |  |  |  |
| 25-31 weeks GA | 21/111 | 19 | 28/127 | 22 | 0.86 | ( | 0.52 | - | 1.42 | ) |
| 28-31 weeks GA | 60/407 | 15 | 45/366 | 12 | 1.20 | ( | 0.84 | - | 1.72 | ) |
| 32-36 weeks GA | 61/2607 | 2.3 | 62/2604 | 2.4 | 0.98 | ( | 0.69 | - | 1.39 | ) |
| 37-42 weeks GA | 916/92 895 | 0.99 | 629/90 579 | 0.69 | **1.42** | **(** | **1.28** | **-** | **1.57** | **)** |
| Total | 9454/889 243 | 1.06 | 6845/842 228 | 0.81 | **1.31** | **(** | **1.27** | **-** | **1.35** | **)** |
| ^a^RR calculated with fetus at risk approach, meaning the numerator is all alive neonates with morbidity in each stratum and the denominator all alive neonates in each birthweight category  GA, gestational age; SGA, small for gestational age; LGA, large for gestational age; RR, relative risk ratio; CI, confidence interval | | | | | | | | | | |

| **Table S8.** Intracranial hemorrhage rate in males and females by birthweight percentiles and gestational age category. | | | | | | | | | | |
| --- | --- | --- | --- | --- | --- | --- | --- | --- | --- | --- |
|  | Male infants |  | Female infants |  |  |  |  |  |  |  |
|  | (n=895 272) | % | (n=847 559) | % | RR^a^ |  | (95%CI) | | |  |
| Birthweight <p10 (SGA) | |  |  |  |  |  |  |  |  |  |
| 25-27 weeks GA | 14/121 | 12 | 6/56 | 11 | 1.08 | ( | 0.44 | - | 2.66 | ) |
| 28-31 weeks GA | 59/475 | 12 | 35/277 | 13 | 0.98 | ( | 0.66 | - | 1.45 | ) |
| 32-36 weeks GA | 43/4431 | 0.97 | 39/3569 | 1.1 | 0.89 | ( | 0.58 | - | 1.37 | ) |
| 37-42 weeks GA | 47/72 340 | 0.06 | 39/70 122 | 0.06 | 1.17 | ( | 0.76 | - | 1.79 | ) |
| Birthweight p10-p90 (normal weight) | |  |  |  |  |  |  |  |  |  |
| 25-31 weeks GA | 231/807 | 29 | 131/640 | 20 | **1.40** | **(** | **1.16** | **-** | **1.69** | **)** |
| 28-31 weeks GA | 592/4047 | 15 | 370/3099 | 12 | **1.23** | **(** | **1.09** | **-** | **1.38** | **)** |
| 32-36 weeks GA | 208/40 765 | 0.51 | 128/32 186 | 0.40 | **1.28** | **(** | **1.03** | **-** | **1.60** | **)** |
| 37-42 weeks GA | 155/670 237 | 0.02 | 104/638 603 | 0.02 | **1.42** | **(** | **1.11** | **-** | **1.82** | **)** |
| Birthweight ≥p90 (LGA) | |  |  |  |  |  |  |  |  |  |
| 25-31 weeks GA | 32/111 | 29 | 46/127 | 36 | 0.80 | ( | 0.55 | - | 1.16 | ) |
| 28-31 weeks GA | 76/407 | 19 | 45/366 | 12 | **1.52** | **(** | **1.08** | **-** | **2.13** | **)** |
| 32-36 weeks GA | 15/2607 | 0.58 | 12/2604 | 0.46 | 1.25 | ( | 0.59 | - | 2.66 | ) |
| 37-42 weeks GA | 36/92 895 | 0.04 | 26/90 579 | 0.03 | 1.35 | ( | 0.82 | - | 2.24 | ) |
| Total | 1508/889 243 | 0.17 | 981/842 228 | 0.12 | **1.46** | **(** | **1.34** | **-** | **1.58** | **)** |
| ^a^RR calculated with fetus at risk approach, meaning the numerator is all alive neonates with morbidity in each stratum and the denominator all alive neonates in each birthweight category  GA, gestational age; SGA, small for gestational age; LGA, large for gestational age; RR, relative risk ratio; CI, confidence interval | | | | | | | | | | |
